# Adaptation to dietary conditions by trehalose metabolism in *Drosophila*

**DOI:** 10.1038/s41598-017-01754-9

**Published:** 2017-05-09

**Authors:** Tetsuo Yasugi, Takayuki Yamada, Takashi Nishimura

**Affiliations:** 1 Laboratory for Growth Control Signaling, RIKEN Center for Developmental Biology (CDB), 2-2-3 Minatojima-Minamimachi, Chuo-ku, Kobe, Hyogo, 650-0047 Japan; 2 Mathematical Neuroscience Unit, Institute for Frontier Science Initiative, Kanazawa University, 13-1 Takaramachi, Kanazawa, Ishikawa, 920-8640 Japan

## Abstract

Trehalose is a non-reducing disaccharide that serves as the main sugar component of haemolymph in insects. Trehalose hydrolysis enzyme, called trehalase, is highly conserved from bacteria to humans. However, our understanding of the physiological role of trehalase remains incomplete. Here, we analyze the phenotypes of several *Trehalase* (*Treh*) loss-of-function alleles in a comparative manner in *Drosophila*. The previously reported mutant phenotype of *Treh* affecting neuroepithelial stem cell maintenance and differentiation in the optic lobe is caused by second-site alleles in addition to *Treh*. We further report that the survival rate of *Treh* null mutants is significantly influenced by dietary conditions. *Treh* mutant larvae are lethal not only on a low-sugar diet but also under low-protein diet conditions. A reduction in adaptation ability under poor food conditions in *Treh* mutants is mainly caused by the overaccumulation of trehalose rather than the loss of *Treh*, because the additional loss of *Tps1* mitigates the lethal effect of *Treh* mutants. These results demonstrate that proper trehalose metabolism plays a critical role in adaptation under various environmental conditions.

## Introduction

Living organisms can adapt to environmental changes through hormonal regulation and metabolic homeostasis. Sugars are primarily utilized in the metabolic production of ATP and carbon sources. In insects, blood sugar is stored as a non-reducing disaccharide trehalose and maintains glucose at low levels^1–3^. Trehalose is widely utilized as a sugar source in several organisms including bacteria, yeast, fungi, plants, and invertebrates^3, 4^. Because of its inert chemical properties, trehalose has the advantage of protecting organisms against several environmental stressors, such as desiccation and starvation^5–7^.

In *Drosophila*, trehalose is synthesized from glucose by trehalose-6-phosphate (Tre6P) synthase (Tps1) in the fat body and degraded to glucose by trehalase (Treh). Tps1 has two functionally distinct catalytic domains^8, 9^. The N-terminus trehalose-6-phosphate synthase (TPS) domain produces Tre6P from glycose-6-phophate (Glu-6P) and UDP-glucose. The C-terminus Tre6P phosphatase (TPP) domain de-phosphorylates Tre6P to generate trehalose. Treh is produced in two different forms via variations in alternative splicing (Flybase): a putative secreted form (sTreh), with a signal peptide at the N-terminus, and a cytoplasmic form (cTreh), without a signal peptide. The expression of *sTreh* is positively regulated by its own activity, whereas cTreh expression is regulated in a compensatory manner^9^. Tissue-specific expression patterns of two distinct forms suggest the systemic and local requirement of trehalose hydrolysis by Treh^8^. In the central nervous system, Treh is predominantly found in surface glia that forms the blood-brain barrier^10^. The local breakdown of trehalose and the following glycolysis in glia produces alanine and lactate. These C_3_ compounds are further metabolized in neurons, which is essential for neuronal survival^10^.

As in mammals, circulating sugar levels in *Drosophila* are regulated by the action of two endocrine hormones, insulin-like peptides (Dilps) and a glucagon-like peptide (Adipokinetic hormone, Akh). Indeed, feeding on dietary sugar immediately changes the levels of circulating sugar^11^. Elevated circulating glucose is taken up by several tissues in part through the action of insulin signaling, while starvation promotes lipid mobilization, the breakdown of glycogen, and gluconeogenesis partly through the action of glucagon signaling^12–14^. Genetic manipulation of the function of Dilps and Akh changes trehalose and glucose levels in the circulating haemolymph^15, 16^. Therefore, the mobilization of blood trehalose to glucose is critical for metabolic homeostasis. However, the physiological importance of circulating sugar metabolism in the adaptation to fluctuations in nutritional conditions remains unclear.

Recently, three groups, including ours, independently generated and reported the mutant alleles of *Treh*. However, the lethal phase is apparently different between mutant alleles. TALEN-induced null alleles of *Treh* are lethal at the first instar larval stage in homozygosity and in *trans* to a chromosomal deficiency^10^. However, we reported that CRISPR/Cas9-induced null alleles of *Treh* are lethal at the pupal stage in homozygosity and in *trans* to a chromosomal deficiency^9^. The other loss-of-function mutants of *Treh* that have a deletion/insertion within the intron region of the *Treh* gene exhibit disorganization in the optic lobe neuroepithelia in the central nervous system and are lethal at the wandering larval and pupal stages^17^.

Here, we report the genetic characterization of the mutant alleles of *Treh* under identical rearing conditions. We found that the reported mutant phenotype of *Treh* affecting neuroepithelial stem cell maintenance and differentiation in the optic lobe is caused by second-site alleles of *lgl*. We further found that loss of *Treh* leads to food-dependent larval lethality. A reduction in adaptation ability under poor food conditions in *Treh* mutants is caused by the overaccumulation of trehalose rather than the loss of *Treh*, because the additional loss of *Tps1* mitigates the lethal effect of *Treh* mutants. These results demonstrate that trehalose metabolism plays a critical role in adaptation under various nutritional conditions in nature.

## Results

### *Treh* mutations do not cause disorganization of the optic lobe in the central nervous system

**Figure 1:**
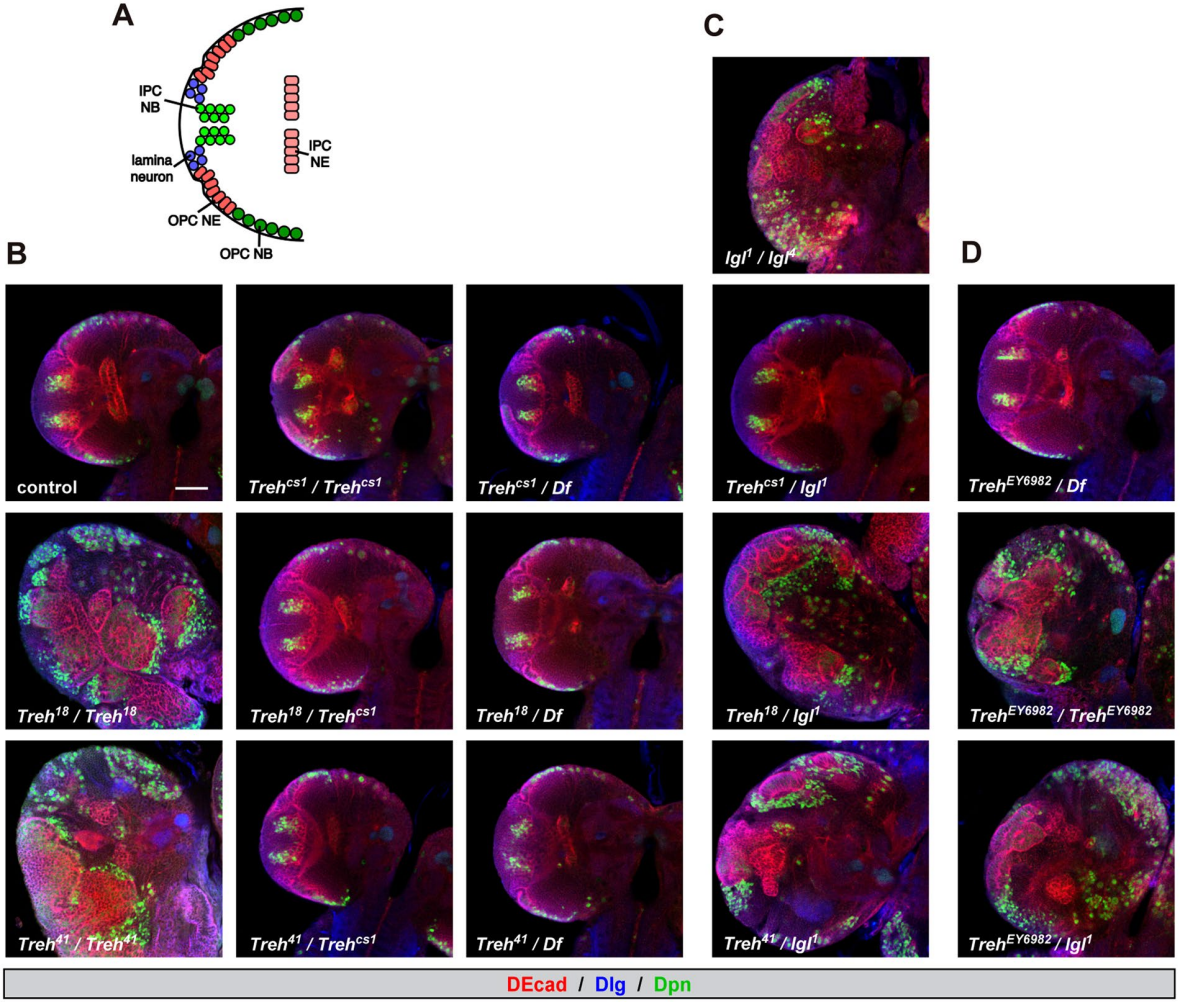
*Treh* mutants do not cause disorganization of the optic lobe in the central nervous system. (**A**) Schematic diagram of the frontal view of the larval optic lobe. Lamina neuron, OPC neuroepithelial cell (OPC NE), OPC neuroblast (OPC NE), IPC neuroepithelial cell (IPC NE), and IPC neuroblast (IPC NB) are shown. (**B**) *Treh* ^*cs1*^ null mutants do not cause disorganization of the optic lobe in the central nervous system in homozygotes and *in trans* to a chromosomal deficiency. Wandering late-third instar larvae were stained for Deadpan (Dpn, green) to label neuroblasts and for Discs large (Dlg, blue) and DE-Cadherin (DE-cad, red) to label epithelial cells. Single medial confocal sections of the optic lobe for each genotype are shown. (**C**) Second site mutation(s) in the *lgl* locus is responsible for the disorganization phenotype of *Treh* ^*18*^ and *Treh* ^*41*^ mutants in the optic lobe neuroepithelia. (**D**) Original *P*-element insertion allele of *Treh* ^*18*^ and *Treh* ^*41*^ retains second site mutation(s) in the *lgl* locus. Scale bars = 50 μm.

The central nervous system is an important tissue where circulating sugar mostly contributes to energy production. There are two proliferation centers in the larval optic lobe, namely, the outer proliferation center (OPC) and the inner proliferation center (IPC) (Fig. 1A)^18, 19^. OPC neuroepithelial cells differentiate into OPC neuroblasts and lamina neurons. IPC neuroepithelial cells are located in the inner and proximal side of the optic lobe and give rise to IPC neuroblasts, which are located on the distal side^19, 20^. It has been reported that *Treh* mutants exhibit disorganization of the optic lobe neuroepithelia in the central nervous system^17^. This phenotype is likely independent of its catalytic activity, because the increase in dietary glucose levels fails to rescue the mutant phenotype^17^.

Unlike the previous report, however, we found that CRISPR/Cas9-induced null alleles of *Treh*, named *Treh* ^*cs1*^, did not display disorganization of the optic lobe neuroepithelia at the wandering third instar larval stage (Fig. 1B). Homozygous mutants of *Treh* ^*18*^ and *Treh* ^*41*^ that were used in the previous report indeed exhibited severe disorganization of the neuroepithelial cells (E-cadherin+ region) and medulla neuroblasts (Dpn+ region). However, transheterozygotes of *Treh* ^*18*^ or *Treh* ^*41*^ over the deficiency allele lacking the *Treh* locus or *Treh* ^*cs1*^ had no obvious phenotype in the optic lobe neuroepithelia. These results suggest that second site mutation(s) are responsible for the observed defects in the optic lobe. Based on the morphological defects and the origin of the mutation on the second chromosome, we tested the contribution of the *lgl* gene locus. Transheterozygotes of *lgl* ^*1*^ over *Treh* ^*18*^ *or Treh* ^*41*^, but not over *Treh* ^*cs1*^, displayed the overgrowth defects (Fig. 1C), indicating that second site mutation(s) are in the *lgl* locus. A high frequency second-site mutation in the *lgl* locus has been reported in the second chromosome stocks^21^. The original *P*-element insertion allele of *Treh* ^*18*^ and *Treh* ^*41*^ that was used for imprecise excision (*Treh* ^*EY6928*^) displayed the identical phenotype (Fig. 1D), indicating that the original *P*-element allele retains a mutation in the *lgl* locus.

**Figure 2:**
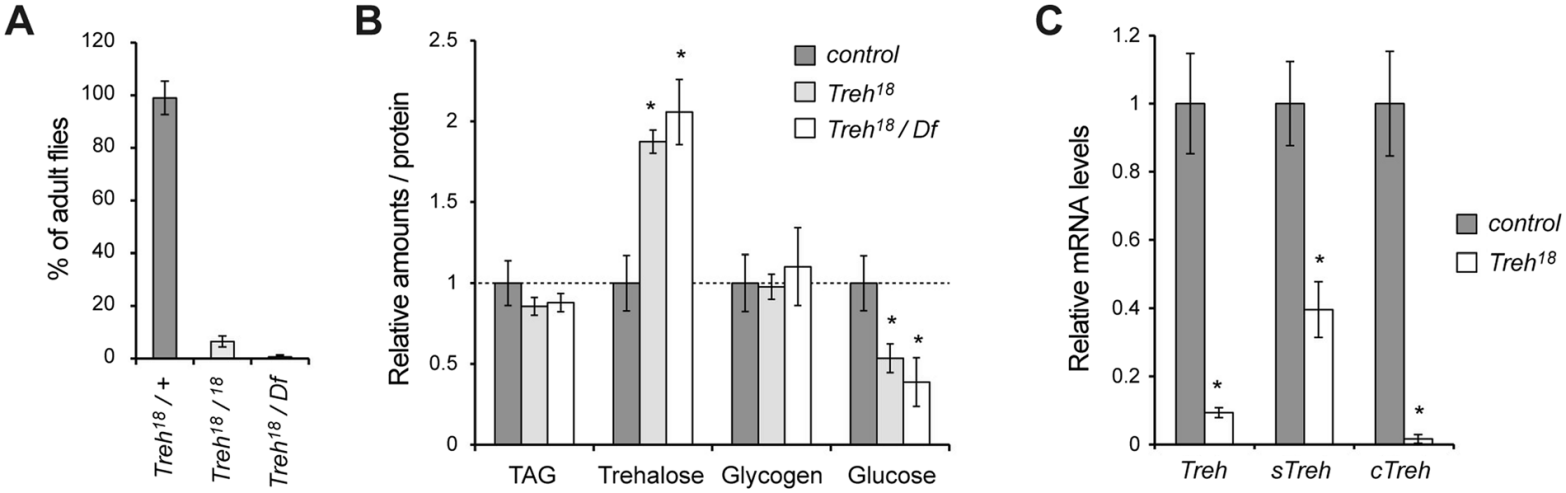
*Treh* ^*18*^ is a hypomorphic allele of *Treh*. (**A**) Backcrossed *Treh* ^*18*^ mutants exhibit pupal lethality with few escapers. The percentage of adult flies was determined by the ratio to flies with a balancer chromosome in each vial. Values shown are means and SEM. n = 6. (**B**) *Treh* ^*18*^ mutants increase trehalose levels and decrease glucose levels in late third instar larvae. Each value was normalized by protein levels and further normalized according to the level in the control larvae. (**C**) *Treh* transcript levels were analyzed by qRT-PCR at the mid third instar stage. Values are means and SD. n = 3 (**B**,**C**). *p < 0.01; (**B**) one-way ANOVA with Dunnett’s post hoc test, (**C**) two-tailed Student’s *t*-test.

**Figure 3:**
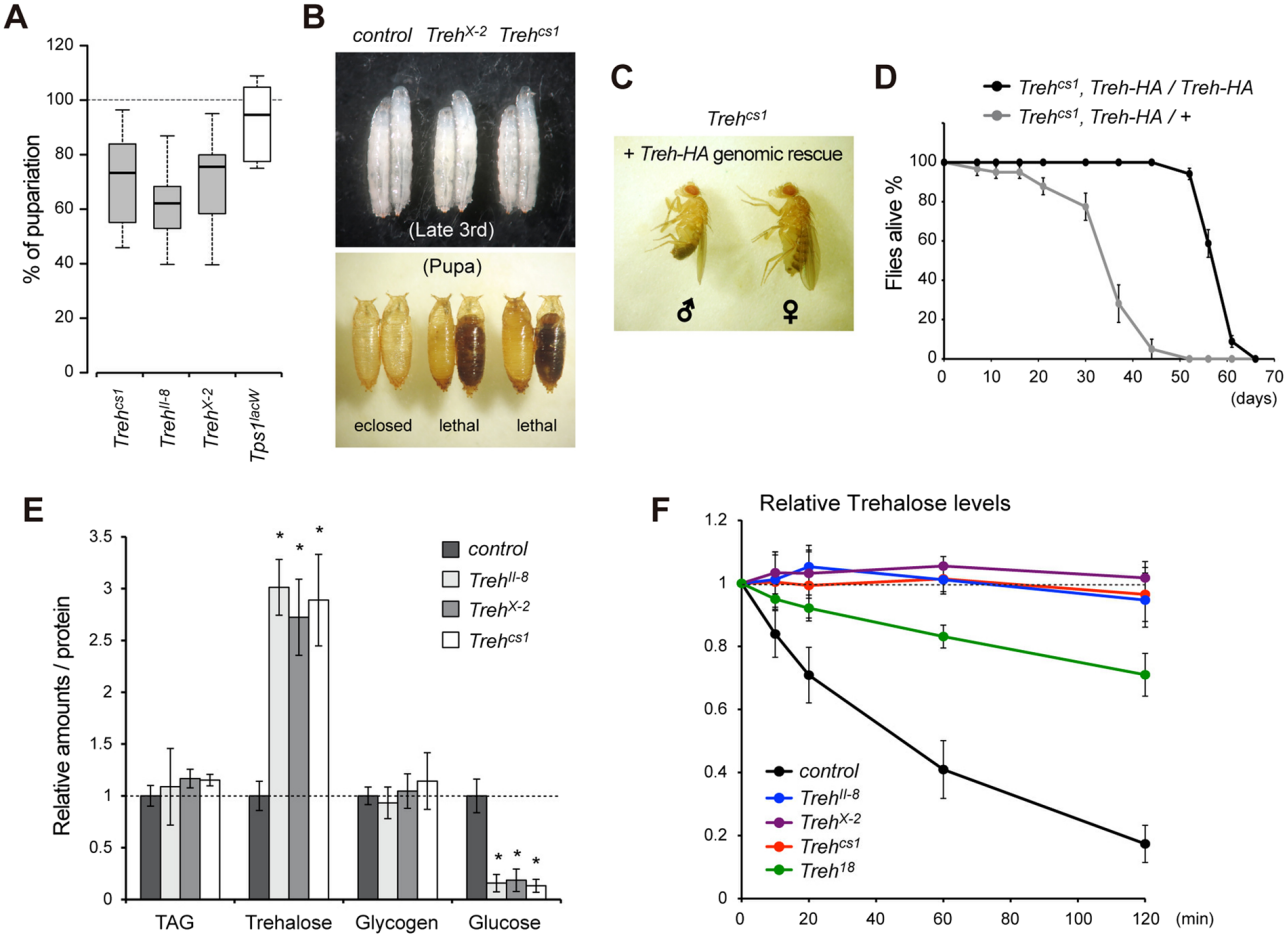
*Treh* ^*II-8*^ and *Treh* ^*X-2*^ mutants are indistinguishable from *Treh* ^*cs1*^ mutants. (**A**) The majority of *Treh* ^*II-8*^ and *Treh* ^*X-2*^ mutants are lethal at the pupal stage. Percentages of homozygous mutant pupae were determined by the ratio to heterozygous mutants in each vial. No statistical significances were detected by one-way ANOVA with Tukey’s post hoc test (p < 0.01). n = 6. (**B**) *Treh* ^*X-2*^ and *Treh* ^*cs1*^ mutants at late-third larval and pupal stages are shown. (**C**) One copy of the genomic rescue construct of *Treh-HA* rescues the pupal lethality of *Treh* ^*cs1*^ homozygous mutants. (**D**) Survival of male flies with one copy or two copies of the genomic rescue constructs of *Treh-HA* in a *Treh* ^*cs1*^ homozygous mutant background. Values shown are means and SEM. n = 4. (**E**) *Treh* ^*II-8*^ and *Treh* ^*X-2*^ mutants exhibit the identical phenotype to *Treh* ^*cs1*^, as revealed by the increase in trehalose levels and decrease in glucose levels in late third instar larvae. Each value was normalized by protein levels and further normalized according to the levels in the control larvae. (**F**) Trehalase activity was measured in larval homogenates. Relative levels of trehalose are shown for each mutant. Values are means and SD. n = 4 (**E**,**F**). *p < 0.01; one-way ANOVA with Dunnett’s post hoc test.

It should be noted, however, that transheterozygotes of *Treh* ^*18*^ *or Treh* ^*41*^ over the deficiency displayed pupal lethality (data not shown). We found that a backcrossed *Treh* ^*18*^ allele without *lgl* mutation(s) displayed no overgrowth in the optic lobe (data not shown) but exhibited pupal lethality with some escapers (Fig. 2A). Indeed, homozygotes of *Treh* ^*18*^ increased the levels of trehalose; however, the degree was less than that of *Treh* ^*cs1*^ (Figs 2B and 3E). In addition, *Treh* ^*18*^ decreased the level of glucose but did not affect the levels of TAG and glycogen. These results are consistent with our previous report^9^. Because both *Treh* ^*18*^ and *Treh* ^*41*^ have a deletion/insertion within the intron region of the *Treh* gene, the hypomorphic phenotype of *Treh* ^*18*^ and *Treh* ^*41*^ is likely due to the reduced transcription and/or alterations of *Treh* mRNA, as reported^17^. Consistent with the pupal lethal phenotype, *Treh* ^*18*^ significantly reduced the expression of c*Treh* in addition to the reduction in *sTreh* (Fig. 2C). These results further demonstrate that *Treh* ^*18*^ is a hypomorphic allele of *Treh*. The mechanism underlying the reduced gene expression due to small changes in the intron remains unknown. The sequence around the small deletion/insertion may contain a critical enhancer region that drives the expression of *Treh*. An alternative possibility is that the sequence change in the intron affects splicing and thereby produces unusual transcripts.

### *Treh* null mutants can survive the larval stage and are lethal during the pupal phase

It has been reported that TALEN-induced null alleles of *Treh*, named *Treh* ^*II-8*^, *Treh* ^*IV-9*^, and *Treh* ^*X-2*^, are lethal in first instar larvae in homozygosity and in *trans* to a chromosomal deficiency^10^. The authors further show that the homozygous mutant lethality can be rescued only when two copies of a genomic rescue construct exist, probably because of the lack of sequences that induce high levels of expression^10^. These results contradict our previous report showing that *Treh* ^*cs1*^ mutants are lethal at the pupal stage in homozygosity and in *trans* to a chromosomal deficiency^9^. To clarify the discrepancy of the lethal stages in *Treh* null mutants, lethal stages of the available *Treh* mutants were compared under identical food conditions. We found that all *Treh* mutant alleles survived the larval stage and died during the pupal stage under our experimental conditions (Fig. 3A,B). However, the number of homozygous mutant pupae compared to heterozygous mutants showed variation between vials, suggesting that there is some larval lethality before the pupal stage. In addition, one copy of the genomic rescue construct was sufficient to fully rescue the pupal lethality of *Treh* ^*cs1*^ mutants (Fig. 3C). These adult flies appeared to be unhealthy with short lifespans compared to those with two copies of the rescue constructs (Fig. 3D). These results suggest that one copy of the construct is sufficient to rescue larval and pupal lethality, but it is not enough to rescue adult lifespan under our experimental conditions. Consistent with this finding, it has been reported that glia-specific knockdown of *Treh* in adults leads to a lifespan reduction^10^. We further confirmed that all *Treh* null alleles increased trehalose levels and reduced glucose levels in a comparable manner (Fig. 3E). In addition, we failed to detect any trehalose hydrolysis activity in the homogenates of these mutants at the wandering stage (Fig. 3F). On the other hand, weak trehalose hydrolysis activity was detected in the homogenates of *Treh* ^*18*^ mutants. These results indicate that all *Treh* null alleles are indistinguishable from each other.

### Survival and growth of *Treh* mutants are highly dependent on dietary conditions

**Figure 4:**
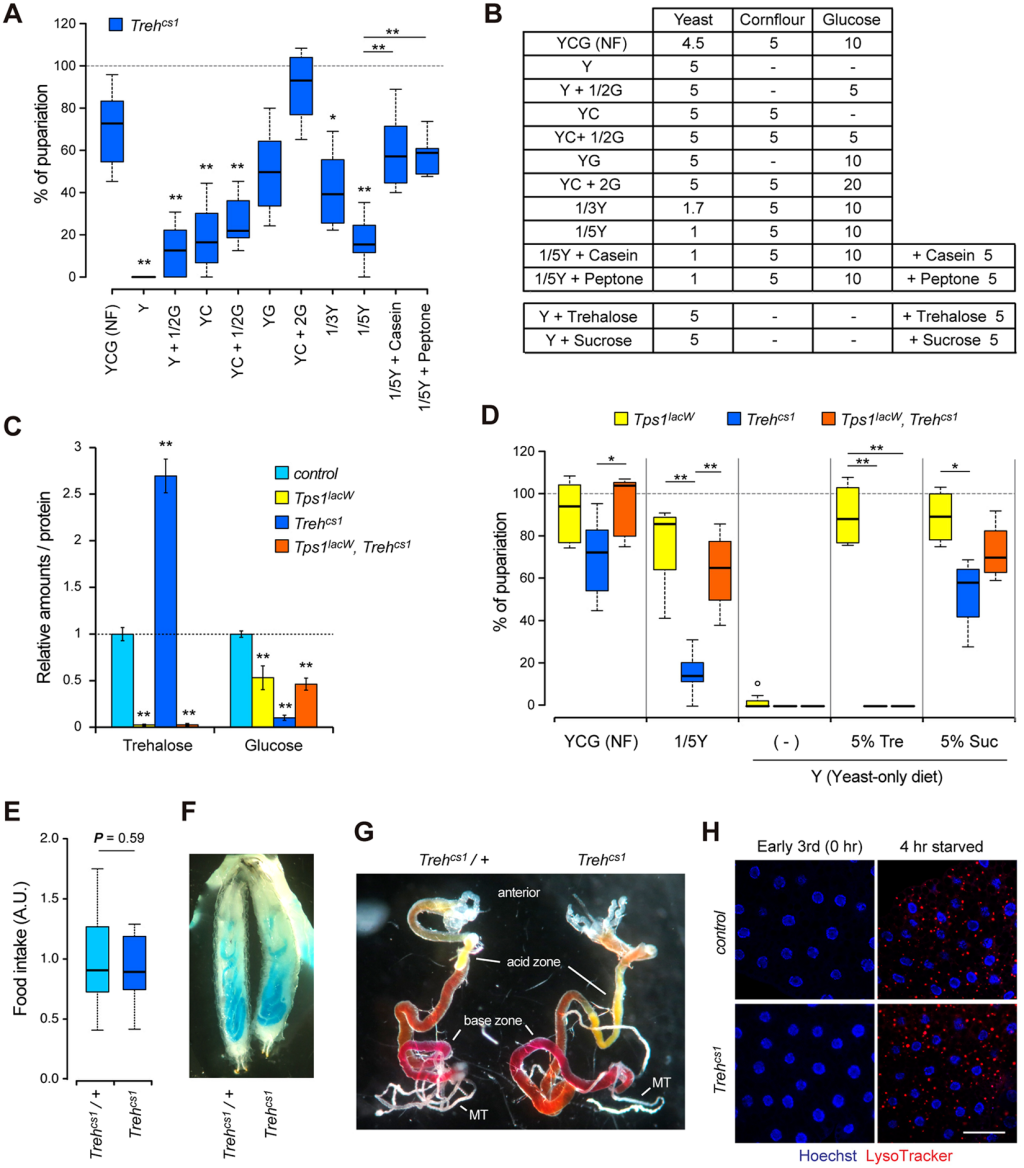
Impact of dietary conditions on the survival of *Treh* mutant larvae. (**A**) *Treh* mutants are sensitive to low-glucose and low-protein dietary conditions. Percentages of homozygous mutant pupae were determined by the ratio to heterozygous mutants in each vial. (**B**) Food compositions used in (**A** and **D**). Values are g per 100 ml. (**C**) The amounts of trehalose and glucose were analyzed in early-third instar larvae. Each value was normalized by protein levels and further normalized according to the level in the control larvae. Values shown are means and SD. (**D**) The pupariation rate of *Tps1* and *Treh* single mutants and *Tps1*, *Treh* double mutants under various dietary conditions. Overaccumulation of trehalose rather than the loss of *Treh* causes deleterious effects on the adaptation to poor food conditions. Tre, trehalose; Suc, sucrose. (**E**) *Treh* mutants exhibit normal feeding behaviour. Food intake levels are evaluated by the rate of blue food ingestion at early third-instar larvae. (**F**) *Treh* mutants retain the epithelial integrity in the midgut. Leakage of ingested blue food into body cavity was tested. (**G**) *Treh* mutants have the normal segmented intraluminal pH zones in the midgut. Mid third instar larvae were fed with food containing phenol red dye to detect luminal pH. Phenol red changes from red to yellow at pH < 3 (acid zone) and to dark pink at pH > 10 (base zone). MT, Malpighian tubules. (**H**) *Treh* mutants exhibit no abnormality of autophagy in the fat body under fed and starved conditions. Dissected fat body at early third instar larvae were stained for LysoTracker (red) and Hoechst (blue) to label nuclei. Scale bar = 50 μm. n = 6~12 (**A**,**D**), n = 4 (**C**), or n = 10 (**E**). **p < 0.01, *p < 0.05; (**A**,**C**,**D**) one-way ANOVA with Tukey’s post hoc test, (**E**) two-tailed Student’s *t*-test.

The above results raise the possibility that the difference in the lethality of *Treh* null mutants in published studies is caused by the difference in dietary conditions. To support this idea, it has been reported that dietary conditions affect the lethality of mutants involved in energy metabolism^22–25^. Indeed, *Tps1* mutants lacking trehalose are highly sensitive to dietary conditions in terms of their survival and body growth^8^. Therefore, we next analyzed the survival rate of *Treh* ^*cs1*^ mutants under several dietary sugar conditions. Consistent with the results for the *Tps1* mutants, *Treh* ^*cs1*^ mutants all died during the larval period under low dietary sugar conditions (Fig. 4A,B). It should be noted that wild-type flies and heterozygous mutants are fully viable under these conditions. We found a clear correlation between the survival rate of *Treh* ^*cs1*^ mutant larvae and the amounts of dietary sugar in food. A high-sugar diet, such as 20% glucose, is known to impair larval growth and cause insulin-resistant phenotypes^26, 27^. Such high-sugar diets further improve the survival rate of *Treh* ^*cs1*^ mutant larvae compared with those under normal food conditions. Of note, none of the food conditions overcame the pupal lethality of *Treh* ^*cs1*^ mutants. The lethality in *Treh* mutants was more severe than that of *Tps1* mutants under identical food conditions, as previously reported^8^, indicating that *Treh* mutants require a higher dose of dietary sugar than *Tps1* mutants for their survival.

In addition to dietary sugar, we found that reducing the yeast content in food significantly lowered the survival rate of *Treh* mutants (Fig. 4A,B). Dry yeast in fly food has been recognized as a major source of protein and amino acids, although it contains many other metabolites. To examine whether the larval lethality of *Treh* mutants is due to the lack of dietary protein or other metabolites, we tested the effect of protein supplementation. For this, we used casein and peptone, a mixture of polypeptides and amino acids formed by the partial hydrolysis of animal protein. In both cases, the supplementation of a protein source substantially rescued the survival rate of *Treh* mutants (Fig. 4A). Taken together, these results suggest that dietary conditions significantly affect the survival rate of *Treh* mutants.

### Overaccumulation of trehalose rather than the loss of *Treh* causes deleterious effects on the adaptation to poor food conditions

Our observations demonstrate that *Treh* mutants are more sensitive to dietary conditions than *Tps1* mutants that lack trehalose. The majority of *Treh* mutants are lethal during the larval stage under chronic low-protein diet conditions. This phenotype is specific to *Treh* mutants, because *Tps1* mutant larvae survive to the pupal stage at a rate almost comparable to wild-type under these conditions^8^. Because *Treh* mutants retain high levels of trehalose in circulation, we hypothesized that the accumulation of trehalose rather than the loss of *Treh* results in lowering the adaption to poor food conditions. To address this possibility, we next generated double mutants for *Tps1* and *Treh* to cancel out the accumulation of trehalose. As expected, the increase of trehalose in *Treh* mutants was completely canceled in *Tps1*, *Treh* double mutants (Fig. 4C). In addition, the significant reduction of glucose in *Treh* mutants was also recovered to some extent in the double mutants. We found that *Tps1*, *Treh* double mutants were lethal at the pupal stage under normal food conditions (Fig. 4D). The lethal effect of *Treh* mutants under low dietary protein conditions was largely suppressed by the additional loss of *Tps1*. These results suggest that the reduction in adaptation ability to low dietary protein in *Treh* mutants is mainly caused by the overaccumulation of trehalose rather than the loss of *Treh*.

We further asked whether the oral administration of trehalose affects the viability of *Tps1* and *Treh* mutants. We found that the addition of trehalose to a yeast-only diet fully rescued the survival and growth of *Tps1* mutant larvae (Fig. 4B,D). However, dietary trehalose failed to rescue the survival of *Treh* mutants and *Tps1*, *Treh* double mutants. Because *sTreh* is highly expressed in the midgut^8^, these results indicate that the utilization of trehalose as dietary sugar requires the function of Treh. On the other hand, the addition of sucrose rescued the survival of all these mutants until the pupal stage. It should be noted that the addition of trehalose or sucrose to the diet is not sufficient to rescue the internal trehalose levels and pupal lethality in *Tps1* mutants (data not shown)^8^.

*Treh* mutants retain higher levels of circulating trehalose, whereas the mutants retain a very low level of free glucose. In addition, *Treh* mutants increase the haemolymph water volume at the expense of the reduction of intracellular fluid^9^. Many animals, including insects, have evolved an osmoregulation system under strict homeostatic control and thereby regulate food and water consumption based on internal nutrient abundance^28–30^. Consistently, *Treh* is highly expressed in the midgut and the Malpighian (renal) tubules during the larval period^8^. It is possible that *Treh* mutants impair feeding behaviour and/or reduce the food absorption rate, resulting in the lethal effect observed under poor food conditions. Indeed, the knockdown of *trehalase* in potato beetle Leptinotarsa larvae has been reported to reduce food consumption^31^. However, we found that *Treh* mutant larvae showed a normal feeding rate compared with control larvae at the early third instar (Fig. 4E). The intestinal integrity of midgut was intact in *Treh* mutants (Fig. 4F). The larval midgut is divided into several functional segments: an anterior neutral zone, a short acid-secreting middle segment, and a long posterior segment that secretes base into the lumen^32, 33^. *Treh* mutants did not display abnormalities in the transport of acids and bases across cell membranes as judged by a pH indicator dye, phenol red (Fig. 4G). These results imply that low adaptation to poor food conditions is unlikely due to the decrease in appetite and the defect in the midgut.

It has been reported that trehalose acts as an inhibitor of the glucose transporter and induces autophagy by depleting cellular energy^34–37^. The reported IC_50_ of the inhibitory effects by trehalose in the mammalian cell culture system is close to the *in vivo* concentration of trehalose in *Treh* mutants^38^. To examine whether the depletion of cellular energy resulted in the induction of autophagy occurs in *Treh* mutants, we next analyzed autophagosome formation in *Treh* mutants. Lysotracker staining revealed that autophagy was not observed in the *Treh* mutant fat body under fed conditions (Fig. 4H). As expected, autophagy was induced shortly after starvation in both control and *Treh* mutants. Similar results are essentially observed in *Tps1* mutants^8^. Taken together, these results demonstrate that *Treh* mutants do not suffer from a reduction in cellular energy and that overaccumulation of trehalose itself causes deleterious effects on the maintenance of homeostasis under poor dietary conditions.

## Discussion

In this study, we have reported the characterization of several mutant alleles of *Treh* under identical rearing conditions. Our observations reveal that the survival rate of *Treh* mutants is significantly influenced by dietary sugar content. Both *Tps1* mutants and *Treh* mutants significantly reduce free glucose levels, suggesting that trehalose in circulation is continuously turned over to maintain available glucose levels. It has been reported that the amount of trehalose is rather stable compared to that of glucose shortly after food intake^11^. In this sense, the production of trehalose from dietary sugar in the fat body appears to be critical for buffering the fluctuation of sugar levels in the body on a long-term basis. The requirement of dietary sugar in both *Treh* and *Tps1* mutants suggests that trehalose metabolism as the main haemolymph sugar plays a pivotal role in systemic energy homeostasis.

We show that the reduction in adaptability to low protein content in *Treh* mutants is caused by the overaccumulation of trehalose rather than the loss of *Treh*. It has been reported that trehalose acts as an inhibitor of the glucose transporter and induces autophagy by depleting cellular energy in mammals^34–37^. This may explain the requirement for a higher dose of dietary sugar and the reduction in adaptability to low protein content in *Treh* mutants. However, to the contrary, trehalose has been reported to inhibit the formation of autophagic vesicles in the *Drosophila* larval fat body by promoting the TOR pathway in *ex vivo* culture conditions^38^. We have shown that neither high nor a lack of trehalose caused by *Treh* or *Tps1* mutants affect autophagy in the larval fat body *in vivo* ^8^. It remains unknown whether trehalose blocks glucose uptake by competing through the glucose transporter in *Drosophila*, because the *in vivo* concentration of circulating trehalose is relatively high. Besides the glucose transport, locomotion behavior in *Treh* mutant larvae looked normal (data not shown), suggesting that body wall muscles function well. We did not observe morphological defects in the Malpighian tubules in *Treh* mutants (data not shown). However, it is possible that *Treh* mutants have a functional deficit in the Malpighian tubules and that may relate to the observed lethality.

Trehalose is synthesized by two-step reactions. We previously reported that both the TPS and the TPP domains in Tps1 are required for the *de novo* synthesis of trehalose in *Drosophila* ^9^. In addition to the increase in trehalose, Tre6P, the precursor of trehalose in the biosynthetic pathway, may be altered in *Treh* mutants. The increase in Tre6P can be canceled in *Tps1*, *Treh* double mutants, because Tps1 catalyzes the production of Tre6P. Tre6P has been shown to control energy metabolism as a signaling molecule in yeast and plants^39–42^. In plants, Tre6P acts through the inhibition of the serine-threonine protein kinase SnRK1, a plant homologue of animal AMPK. The evolutionarily conserved SnRK1/AMPK is a sensor of energy availability and is activated under conditions of energy depletion and metabolic stress to inhibit growth for cell survival. Tre6P levels are relatively low under normal conditions. Therefore, the alteration in Tre6P levels, in addition to the increase in trehalose caused by deletion of *Treh*, may cause metabolic defects in the fat body. Further analysis will be required to elucidate the cause of lethality in *Treh* mutants under poor diet conditions.

Metabolic parameters are affected not only by genotypes but also by food conditions^43, 44^. Nutritional quality of commercially available dry yeast varies between manufacturers and between batches. A chemically defined standard food would be ideal to compare the mutant phenotype in different laboratories. Chemically defined food or holidic medium sustains whole development in *Drosophila* ^45, 46^. However, the growth rate of larvae is significantly lower than those reared on standard fly food containing yeast. Food composition affects metabolic homeostasis and thereby potentially influences the survival and growth rate. In particular, nutritional quality has a significant impact on mutant flies defective for enzymes involved in metabolic pathways and for endocrine hormones that regulate energy metabolism^22–25^. In contrast to *Tps1* and *Treh* mutants, sugar-dependent lethality has been reported in larvae lacking the conserved carbohydrate response element-binding protein (ChREBP) *Mondo-Mlx* glucose-sensing transcription factors^47^. TGF-β/Activin-like ligand *Daw* mutants also exhibit high-sugar dependent larval lethality^48^. These factors control sugar homeostasis through the regulation of gene expression involved in glycolysis, the TCA cycle, lipogenesis, and β-oxidation^47–49^. Elucidating the precise regulation mechanisms of trehalose metabolism will facilitate our understanding of the adaptation of animals to various environmental conditions.

## Materials and Methods

### *Drosophila* strains

The following stocks were used: *w* ^*1118*^ (used as a control), *Treh* ^*18*^, *Treh* ^*41*^ (from X. Chen and H. Luo), *Treh* ^*II-8*^, *Treh* ^*X-2*^, *Treh-HA* genomic rescue construct (from S. Schirmeier), and *lgl* ^*1*^ and *lgl* ^*4*^ (from F. Matsuzaki). *Tps1* ^*lacW*^ and *Treh* ^*cs*^ have been described previously^8, 9^. *Treh* ^*EY06982*^ and *Df*(*2R*)*Exel6072* (a deficiency for the *Treh* locus) were obtained from the Bloomington *Drosophila* Stock Center. *Treh* ^*18*^ was back-crossed two times with the *w* ^*1118*^ strain and used for experiments shown in Figs 2 and 3.

### Fly food

*Drosophila melanogaster* flies were reared on standard agar-cornmeal media at 25 °C unless otherwise indicated^8, 50^. The detailed food compositions, except preservatives, are shown in Fig. 4B. Casein, peptone, and trehalose were obtained from Wako Chemical, ﻿BD Biosciences, and Hayashibara, Co., respectively. All experiments were conducted under non-crowded conditions. No yeast paste was added to the fly tubes for any of the experiments. The percentage of puparium formation and adult flies was determined by counting homozygotes and heterozygotes in the same vials as an internal control.

### Immunohistochemistry

Larval tissues were dissected in PBS and fixed for 10 min in 3.7% formaldehyde in PBS containing 0.1% Triton X-100 and processed as previously described^51, 52^. The following primary antibodies were used: guinea pig anti-Deadpan (from J. A. Knoblich), mouse anti-Discs large, and rat anti-DEcad2 (from Developmental Studies Hybridoma Bank). Alexa-conjugated secondary antibodies (Invitrogen) were used. The nuclei were stained with Hoechst 33342 (Invitrogen). Images were acquired with a Zeiss LSM700 confocal microscope and were processed in Photoshop (Adobe Systems). Staining of acidic organelles was done as described previously^8^.

### qRT-PCR analysis

qRT-PCR analyses were performed as described previously^50, 51^. The primers used to detect *Treh* (*common*), *sTreh*, *cTreh*, and *rp49* levels were described previously^8, 9^.

### Measurement of protein, TAG, and sugar levels

The measurement of protein, TAG, trehalose, glycogen, and glucose was performed as described previously^8^.

### Trehalase activity assay

Trehalase activity assay was performed as described previously^9^. Two late third instar larvae were rinsed in PBS and homogenized on ice in 100 μl of 10 mM ammonium acetate buffer (pH 5.0) containing 0.1% TritonX-100, 2.5 mM EDTA, and Complete protease inhibitor (Roche). Five μl of the homogenates was mixed on ice with 10 μl of substrate solution (200 ng/μl trehalose and 20 ng/μl mannitol-1-^13^C (SIGMA) as an internal control in 10 mM ammonium acetate, pH 5.0). The reactions were started at 30 °C and stopped after the appropriate times by placing the tubes at 90 °C for 5 min. After cooling to room temperature, the assay reactions were mixed with 85 μl of acetonitrile and cleared by centrifugation, and 20 μl of the supernatant was diluted with 20 μl H_2_O. The amounts of trehalose were quantified by LC-MS/MS.

### Quantification of trehalose and glucose by LC-MS/MS

Chromatographic separation was performed on an ACQUITY BEH Amide column (100 mm × 2.1 mm, 1.7 μm particles, Waters) in combination with a VanGuard precolumn (5 mm × 2.1 mm, 1.7 μm particles) using an Acquity UPLC H-Class System (Waters). Elution was performed at 30 °C under isocratic conditions (0.3 ml/min, 70% acetonitrile and 30% 10 mM ammonium bicarbonate, pH 10.0). The mass spectrometric analysis was performed using a Xevo TQD triple quadrupole mass spectrometer (Waters) coupled with an electro-spray ionization source in negative ion mode. The MRM transitions of *m/z* 341.2 -> 119 and *m/z* 182.1 -> 88.9 were used to quantify trehalose and mannitol-^13^C, respectively. Analytical conditions were optimized using standard solution. Sample concentrations were calculated from the standard curve obtained from serial dilution of each standard. The amounts of trehalose were normalized to the levels of mannitol-1-^13^C and further normalized to the levels at time 0 to determine the relative hydrolysis rate in each mutant. For the quantification of glucose, the MRM transition of *m/z* 179.1 -> 89.0 was used to detect glucose under conditions identical to those described above.

### Food intake assay

Food intake assay was done as described previously^8^. Early third instar larvae were starved for 2 hours on adverse food conditions (0.8% agar in PBS) and then transferred to fresh dye food (0.5% Brilliant Blue FCF) for 20 min. After feeding, the larvae were washed in PBS, dried on tissue paper, and homogenized in 100 μl lysis buffer (6 M guanidine-HCl solution containing 0.1% Triton X-100). After boiling and centrifugation, 2 μl of supernatant was analyzed in a spectrophotometer at 630 nm. For the assessment of midgut pH, mid third instar larvae were fed with food containing 0.2% Phenol Red. After 3 hours, larvae were dissected in PBS and the midgut was photographed under a stereomicroscope (Zeiss) equipped with a digital camera (Canon). For the assessment of midgut integrity, larvae were fed with food containing 0.2% Brilliant Blue FCF for 3 hours. Larvae were observed as above.

### Statistical analysis

Statistical significance was determined by two-tailed Student’s *t*-test, one-way ANOVA with Dunnett’s post hoc test or with Tukey’s post hoc test using GraphPad Prism 6 software. Box plots were drawn online using the BoxPlotR application (http://boxplot.tyerslab.com/). Centerlines show the medians; box limits indicate the 25th and 75th percentiles; whiskers extend 1.5 times the interquartile range from the 25th and 75th percentiles, and outliers are represented by dots.
